# Hardware-Agnostic Imitation Learning Method for Autonomous Ultrasound Scanning Addressing Physical Deployment Discrepancies

**DOI:** 10.3390/s26092804

**Published:** 2026-04-30

**Authors:** Zhuoyang Ma, Jing Xia, Hong Gao, Hongbo Zhu, Yongkang Tang

**Affiliations:** School of Mechanical Engineering, Xi’an University of Science and Technology, Xi’an 710054, China

**Keywords:** autonomous ultrasound scanning, imitation learning, physical-agnostic, cross-instance deployment, waypoint extraction

## Abstract

**Highlights:**

**What are the main findings?**

- A novel physical-parameter-decoupled imitation learning framework is proposed to enable the cross-instance deployment of ultrasound scanning skills across manipulators with objective physical discrepancies.
- The method achieves consistent scanning accuracy and trajectory stability across different physical equipment instances without the need for target-specific retraining.

**What are the implications of the main findings?**

- Research on autonomous ultrasound robots can address the issue of occupational diseases among sonographers, promote the standardization and normalization of ultrasound scanning procedures, and alleviate the global shortage of ultrasound medical personnel.
- The proposed physical-parameter-decoupled end-to-end model overcomes the constraints of low-level kinematic parameters and base coordinate systems. This significantly improves the reliable reusability of expert datasets and skill reproduction efficiency, advancing the broader clinical deployment of autonomous ultrasound robotics.

**Abstract:**

To achieve autonomous ultrasound scanning skill transfer across different physical equipment instances and address the limitations of traditional imitation learning methods—which struggle with cross-instance generalization due to their reliance on specific manipulator parameters—this study proposes a physical-parameter-decoupled imitation learning method based on waypoint representation. This approach utilizes a greedy algorithm to automatically extract key nodes within the task space from expert demonstration trajectories, constructing a trajectory representation decoupled from low-level kinematic parameters and base calibration errors. Simultaneously, a velocity-aware adaptive error precision adjustment mechanism is introduced to dynamically modulate waypoint extraction thresholds, simulating the speed-accuracy strategies employed by sonographers across different scanning phases. Cross-validation across two mainstream generative architectures—Action Chunking Transformer (ACT) and Diffusion Policy—on an offline dataset confirms the plug-and-play capability of waypoint representation in suppressing long-horizon error accumulation, with both architectures achieving significant reductions in prediction errors. For physical deployment, a complete ACT-waypoint system featuring low-level triple safety redundancy was validated. In kidney long-axis standard plane scanning tasks, the system achieved a 92% success rate on the source domain manipulator and maintained an 84% success rate on the target deployment manipulator, despite incompatible low-level kinematic parameters and base coordinates. Force control accuracy remained stable around the target value of 12 N. The results demonstrate that the proposed method effectively overcomes base coordinate and D-H parameter discrepancies to achieve cross-instance zero-shot skill transfer, significantly enhancing the adaptability across physical instances and the scanning success rate of imitation learning models.

## 1. Introduction

Autonomous ultrasound scanning robots have emerged as an effective technical solution to address challenges in ultrasound diagnosis, such as the scarcity of professionals, operator variability, and the uneven distribution of medical resources [1]. As a crucial technical pathway, imitation learning enables robots to learn and autonomously perform ultrasound examinations through expert demonstration [2]. However, data acquisition and skill reproduction in existing imitation learning methods are typically conducted on the same manipulator platform, rendering the resulting models unable to be reused on other manipulators. This not only reduces the cross-instance utilization efficiency of the datasets but also significantly restricts the further development of autonomous ultrasound scanning robots.

Su et al. [3] employed an imitation learning method, utilizing Cartesian poses as model output actions, to collect ultrasound scanning demonstration data on a 6-DOF UR3 manipulator and complete skill reproduction on the same platform. Similarly, the autonomous ultrasound scanning models proposed in [4,5] used Cartesian poses as model output actions to achieve the learning and reproduction of scanning skills on a single manipulator. Although these works have made significant progress, their training and reproduction are characterized by an over-reliance on a single manipulator environment. This severely restricts the model’s generalization capability, making it difficult to address the objective deployment discrepancies encountered in real clinical environments.

The autonomous ultrasound scanning model proposed in [6] utilizes 12 discrete Cartesian actions and a stop signal as model outputs, collecting data and achieving autonomous ultrasound scanning on the same manipulator. Similarly, the autonomous ultrasound scanning models proposed in [7,8] adopt Cartesian pose increments as model outputs, completing both ultrasound scanning data collection and skill reproduction on a single manipulator. By using Cartesian pose increments or discrete Cartesian actions as model outputs, it is theoretically possible to adapt to different equipment instances; however, these studies lack real-world physical validation, making it impossible to determine their transfer performance. Furthermore, due to the absence of a feedback mechanism, relying on Cartesian pose increments or Cartesian actions as model outputs means that minor errors in predicted actions can trigger substantial deviations. This easily causes prediction errors to accumulate quadratically over time (error accumulation), thereby resulting in the typical “compounding error” problem and causing the policy to deviate from the state distribution covered by the demonstration data [9].

In the field of robotic transfer learning, to address the challenge of directly transferring learned skills to other hardware platforms, ref. [10] attempted to achieve cross-manipulator skill transfer through a unified representation space. A policy model is learned within this latent space, where latent actions are subsequently decoded into executable states and actions for the target manipulator, thereby realizing skill transfer. However, the optimal latent space dimension utilized in this method is restricted to four; such low dimensionality is insufficient to adequately represent complex motions in three-dimensional space. Furthermore, its latent dynamics are modeled deterministically, failing to account for stochastic factors universally present in real robotic systems, such as friction and backlash. In highly nonlinear manipulation tasks, this limitation may lead to inadequate control precision, thus restricting its applicability in complex ultrasound scanning scenarios. Similarly, ref. [11] proposed a modular cross-hardware transfer framework. This framework first encodes task states and constructs relative representations via a task module, uniformly mapping the actions of different manipulators into standardized latent variable representations. Subsequently, the latent variables and the intrinsic state of the target deployment manipulator are jointly fed into a robot module to decode and generate action commands tailored to specific hardware platforms. However, this method has been primarily validated on single-step manipulation tasks, such as pushing and picking. Its design assumptions and validation scenarios do not yet encompass complex manipulation tasks characterized by long-horizon dependencies, continuous contact constraints, and stringent safety requirements. Therefore, its applicability in medical robotic applications, such as autonomous ultrasound scanning, remains to be further evaluated.

To circumvent physical parameter deviations across different manipulator instances, refs. [12,13] adopt the Cartesian pose of the end-effector as a unified action representation, utilizing trajectory data from the source manipulator to train a forward dynamics model. By taking the pose and action of the source manipulator as inputs, this model predicts the target pose for the subsequent time step, thereby achieving cross-instance skill transfer. However, this approach relies heavily on high-precision end-effector pose tracking and provides limited support for tasks necessitating precise force control and continuous contact constraints. Consequently, its direct applicability remains restricted in autonomous ultrasound scanning scenarios, which impose stringent requirements on physical safety and imaging quality.

Compared to the aforementioned skill transfer methods, extracting key waypoints from the manipulator’s motion path allows the expert demonstration trajectories to be decoupled into key nodes within the task space. This effectively shortens the length of the decision sequence and significantly mitigates the issue of temporal errors being amplified quadratically during the prediction process. The waypoint-based global trajectory prediction better aligns with the goal-oriented decision-making patterns of human experts in actual operations. Simultaneously, it eliminates the reliance on the hardware-coupled joint space representation, freeing the learning process from the limitations imposed by objective physical deployment discrepancies. Consequently, this significantly enhances the reusability of demonstration data across different physical instances and the overall cross-instance generalization ability of the model.

While existing research by Shi et al. [14] has also employed imitation learning models to extract waypoints from demonstration trajectories—proving its efficacy in reducing trajectory reconstruction errors—its validation has primarily focused on tasks with lower operational complexity, such as sorting and wiping. Such methods are difficult to apply directly to complex ultrasound scanning contexts, which are characterized by stringent precision requirements and dynamic response characteristics. Therefore, further methodological improvements and expansions are imperative for medical robotic scenarios.

**Figure 1:**
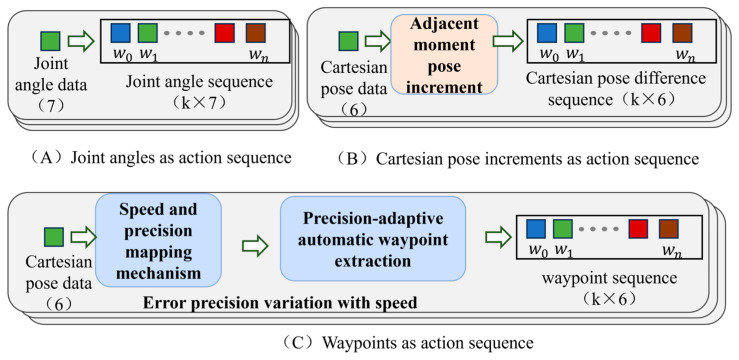
Methods for Action Sequence Extraction of Different Models. (**A**) Joint Angle (**B**) Cartesian Pose Increment (**C**) Waypoint.

This paper proposes a hardware-agnostic imitation learning method addressing physical deployment discrepancies. Based on demonstrations of human kidney long-axis standard plane scanning, this method achieves zero-shot skill transfer without retraining between different equipment instances with objective parameter deviations. Compared with traditional imitation learning methods, the core innovation of this study lies in the representation and processing of demonstration trajectories (as shown in Figure 1). Traditional methods usually adopt joint angle sequences as the action representation of the model [15] (Figure 1A); however, the joint space is tightly bound to the structure and kinematics of a specific manipulator, making it difficult for the model to generalize in real-world deployment environments with low-level physical parameter shifts. Although another category of methods utilizes Cartesian pose increment sequences as action representations (Figure 1B) to bypass the dependence on joint structures, point-by-point prediction continuously amplifies errors in the temporal dimension, making it highly susceptible to significant cumulative deviations. To overcome these limitations, this study introduces waypoint representation to map the complete demonstration trajectory into key nodes in the task space. This enables the model to achieve cross-instance deployment and effectively suppress predictive error accumulation while preserving the action intent and global structure of the trajectory. The main contributions of this paper are as follows:

(1) A hardware-agnostic and algorithm-versatile autonomous ultrasound scanning framework based on waypoint representation is proposed. This representation strategy not only eliminates the influence of kinematic parameter discrepancies and base deployment deviations to enable cross-instance reusability but is also proven to function as a plug-and-play module that consistently reduces long-horizon cumulative errors across different generative backbone networks, such as ACT and Diffusion Policy.
(2) A velocity-aware adaptive waypoint extraction method based on dynamic error adjustment is introduced, allowing the model to focus more on high-precision fine-tuning regions while maintaining scanning efficiency.
(3) A dual-instance validation platform with significant base coordinate and D-H parameter discrepancies is established and the cross-instance universality of the proposed model is verified.

## 2. Materials and Methods

### 2.1. Data Sources

#### 2.1.1. Dataset Construction

**Figure 2:**
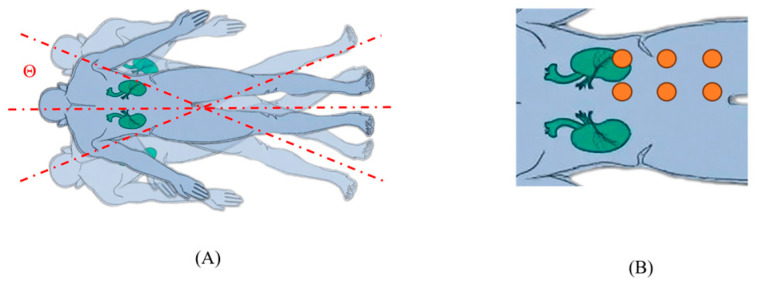
Schematic of human posture variations introduced in the dataset. (**A**) Simulated variations in human posture. (**B**) Initial probe placement points corresponding to positional shifts. In (**A**), the red dashed lines and the symbol Θ represent the central axis and the angular range of the simulated posture variations. In (**B**), the orange circles indicate the discrete initial probe placement points.

This study constructed a dedicated dataset comprising 180 sets of high-quality expert demonstration trajectories, collected from a 24-year-old healthy male volunteer. During the acquisition process, the subject was maintained in a prone position to accurately simulate the clinical posture for dorsal kidney scanning. To validate the model’s kinematic robustness and spatial adaptability to base-to-subject relative shifts, systematic variations in the subject’s relative position and posture were intentionally introduced during the data collection phase. These variations encompassed multi-axis planar translations of the human body (e.g., diagonal shifts) as well as controlled trunk rotations. This approach ensures that the dataset captures a variety of relative spatial configurations rather than a single fixed pose. The specific distribution of pose variations and the initial probe placement points are illustrated in Figure 2.

During data acquisition, the expert physician teleoperated the manipulator to execute the comprehensive clinical workflow, from identifying the initial probe placement point to scanning the kidney long-axis standard plane. The recording session concluded once the target standard plane was clearly visualized in the ultrasound image. The system synchronously logged the end-effector’s Cartesian pose sequence, six-dimensional contact force data, and corresponding ultrasound image frames at 70 ms intervals (i.e., a sampling rate of approximately 14.3 Hz). This sampling rate ensures sufficient data density for capturing fine-grained operations. Consequently, the resulting dataset not only contains the final standard plane images but also fully encapsulates the physician’s dynamic decision-making process, including acoustic window localization, probe orientation adjustments, and dynamic contact force modulation.

#### 2.1.2. Data Acquisition Platform System

**Figure 3:**
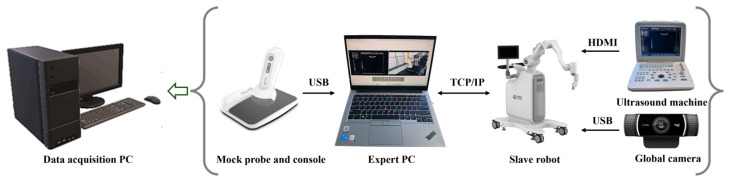
Architecture of the bilateral teleoperated platform for multimodal ultrasound data acquisition.

To acquire clinical data that authentically captures expert manipulation techniques, this study established a bilateral teleoperated ultrasound data acquisition platform (Figure 3). The platform employs a master–slave control architecture comprising three primary subsystems:

(1) Expert Interaction System: This features a customized force-feedback dummy ultrasound probe and an expert console. The expert-end PC processes motion commands in real-time via the TCP/IP protocol and maps them to the patient-end manipulator. Concurrently, it receives visual and haptic feedback from the slave system, establishing an immersive teleoperation environment.
(2) Patient-End Execution System: A 7-DOF HEBIN-RUS-T7 collaborative manipulator serves as the execution mechanism. The end-effector integrates a KW-6065W 6-axis force/torque sensor and a custom 3D-printed probe holder, enabling high-fidelity perception of dynamic contact forces between the probe and the human skin.
(3) Multimodal Imaging and Transmission System: This utilizes a Sonostar SS-6 wireless convex array probe for real-time ultrasound imaging, which is transmitted to the host computer via an HDMI interface. Simultaneously, a Logitech C922 (Logitech, Lausanne, Switzerland) global camera captures the patient-end environmental video stream, routing it back to the expert console via USB. This ensures the physician maintains a comprehensive operational field of view alongside real-time anatomical structural feedback.

### 2.2. Hardware-Agnostic Imitation Learning Framework

**Figure 4:**
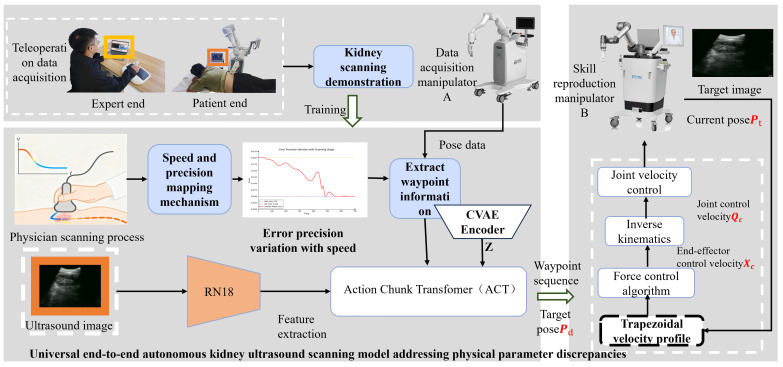
The technical route of this research is primarily divided into teleoperation data acquisition and autonomous ultrasound robot control; the blue shaded areas highlight the main contributions of this paper.

The technical route of this study is illustrated in Figure 4, aiming to enable the reliable cross-instance reproduction of data acquired by a source domain device (Manipulator A) on a target deployment manipulator (Manipulator B) that exhibits base calibration and low-level kinematic parameter discrepancies. The framework comprises three primary components. First, in the ultrasound data acquisition phase, expert physicians record teleoperated demonstrations of in vivo human kidney long-axis standard plane scanning. Second, the data processing and model training phase constructs a velocity-aware mapping function for waypoint extraction precision by analyzing the relationship between probe velocity and operational precision during the expert’s scanning process. Waypoint information is extracted from the demonstration trajectories, decoupling it from specific physical parameter bindings. These waypoint sequences, alongside feature-extracted ultrasound images, are fed into a generative model for training. While the Action Chunking Transformer (ACT) is employed as the foundational backbone network for physical deployment, the waypoint strategy is designed as a plug-and-play module generalizable to other architectures. The model outputs a sequence of waypoints representing target poses in the task space. Third, in the robotic control phase, after performing interpolation between the predicted target poses and the current pose of the target manipulator, joint velocity parameters are calculated using low-level force control algorithms and inverse kinematics (IK). Finally, joint control commands drive the target manipulator to autonomously scan until the target image is reached, effectively isolating errors resulting from physical parameter shifts at the underlying control level.

#### 2.2.1. Waypoint Representation and Network Structure

To achieve imitation learning for autonomous ultrasound scanning, this paper proposes a waypoint representation model demonstrating cross-instance universality (exemplified by the Transformer architecture employed in our physical deployment). The core concept of this model is to decouple the joint action representation—which is tightly bound to low-level kinematic parameters in traditional imitation learning—into a sequence of key waypoints within the task space. Consequently, the learning process no longer relies on the demonstrator’s specific base calibration and D-H parameters, significantly enhancing the reusability of skills across different equipment instances. Note: As a plug-and-play front-end module, this waypoint extraction and representation logic is equally applicable to other generative architectures, such as Diffusion Policy.

**Figure 5:**
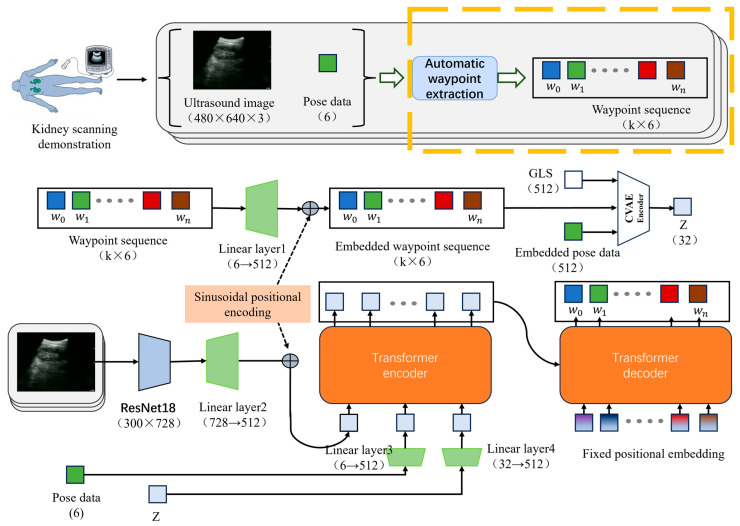
Schematic diagram of an autonomous ultrasound scanning model based on imitation learning.

The overall model architecture is illustrated in Figure 5. Initially, teleoperation is utilized to collect kidney scanning demonstration data, acquiring synchronized ultrasound images and Cartesian pose sequences. Subsequently, key waypoints are extracted from the pose sequences to construct a universal waypoint representation that can be shared across different physical instances. The 6-DOF pose vector of each waypoint is projected into a 512-dimensional feature space via a linear layer (Linear-1) and fed into the encoder of a Conditional Variational Autoencoder (CVAE)—alongside a classification token (CLS) and the current Cartesian pose—to generate a latent variable Z that encapsulates the physician’s operational style.

After extracting features from the ultrasound images using ResNet-18, a 300 × 512-dimensional image feature sequence is obtained through linear transformation. This sequence is input into the Transformer encoder alongside the Cartesian pose features and the style variable Z. The sequence tokens output by the encoder thus integrate a global context encompassing visual information, manipulator states, and operational style.

Finally, aided by timestep positional encoding, the Transformer decoder generates an “Action Chunk” containing k steps of future waypoint predictions. To address the inter-frame trajectory jitter that may arise when the Transformer decoder relies solely on positional encoding in complex long-horizon tasks, this study further introduces a Temporal Ensembling mechanism at the model output as an implicit temporal constraint. Specifically, as the inference window slides along the temporal axis, the waypoint commands for the same physical timestep are repeatedly overwritten by multiple adjacent prediction chunks. At the execution layer, the system applies an Exponential Weighted Moving Average (EWMA) to these overlapping predicted waypoints, assigning higher confidence weights to predictions temporally closer to the current moment.

Following this temporal ensembling smoothing, the resulting waypoint sequence forms a universal task-space trajectory capable of overcoming low-level parameter discrepancies, thereby achieving highly stable, end-to-end modeling of expert demonstration behaviors. Ultimately, the waypoint-based task-space representation adopted in this study effectively circumvents the limitations of traditional incremental and joint-space methods regarding cross-instance deployment and long-horizon error accumulation.

#### 2.2.2. Adaptive Waypoint Extraction Algorithm

To provide the network with high-quality training targets, continuous expert demonstration trajectories must be discretized into sparse sequences of key waypoints. Traditional fixed-threshold sampling struggles to accommodate the dual requirements of “coarse positioning” and “fine-tuning” inherent in ultrasound scanning tasks. To address this limitation, this study proposes an adaptive waypoint extraction algorithm that integrates a greedy strategy with a velocity-aware mechanism.

(1) Automatic Waypoint Extraction

The core objective of automatic waypoint extraction is to divide the original demonstration trajectory into several key waypoints, thereby replacing the complete trajectory with a more compact waypoint sequence, reducing the length of the decision sequence, and mitigating the impact of error accumulation. For this purpose, this paper adopts a waypoint selection method based on a greedy strategy, selecting the waypoint with the minimum current error as the local optimal solution at each step. Compared with other trajectory simplification methods, the greedy algorithm is simple to implement with low computational overhead, and typically controls the compression result directly through an error threshold [16]; therefore, it is highly sensitive to threshold changes, enabling it to better adapt to the dynamic coupling relationship between speed and precision during the ultrasound scanning process.

Based on the above ideas, the overall process of the adaptive waypoint extraction algorithm proposed in this paper is as follows: to formalize the automatic waypoint extraction process, we model it as an optimization problem solved step-by-step. For an expert demonstration trajectory $X=\left[x_{0},x_{1},\ldots ,x_{N}\right]$ our goal is to select the shortest subsequence $W=\left\{w_{1},w_{2},\ldots ,w_{k}\right\},K\ll T$ from it without human intervention to minimize the total error between the original trajectory and the interpolated trajectory. The objective is to select as few waypoints as possible while ensuring the trajectory approximates the original one.

(1) $$\min_{W}\sum_{i=1}^{N}{\left\|x_{i}-X_{\mathrm{interp},i}\right\|}^{2}\;s.t.\;|W|\to \min,$$

where $X_{\mathrm{interp}}$ represents the trajectory point obtained by linear interpolation of the selected waypoints. $\lambda_{i}$ is a weight coefficient used to control whether a given point is the selected waypoint, and if so $\lambda_{i}=0$.

Upon completing waypoint extraction, the model’s training objective transitions from predicting the next proprioceptive state—as seen in traditional methods—to predicting the position of the subsequent key waypoint. This paradigm shift effectively reduces the trajectory length, compressing the original long-horizon sequence into a concise sequence of waypoints. Consequently, this diminishes training complexity and significantly mitigates error accumulation induced by long-horizon dependencies. Simultaneously, because waypoints are defined within the absolute task space rather than the hardware-coupled joint space, the model circumvents reliance on the demonstrator’s specific base calibration and low-level kinematic parameters. This endows the model with the capability for reliable reuse across physical instances. Through this global mapping mechanism from the current state to the next task-critical node, the model effectively overcomes the limitations when facing physical deployment discrepancies inherent in traditional point-by-point incremental control. Ultimately, this significantly enhances the system’s stability, reproducibility, and cross-instance adaptability when confronting objective physical parameter deviations across different equipment instances.

(2) Adaptive error precision adjustment based on velocity variation

To enhance the adaptability and reproducibility of the waypoint extraction algorithm across different scanning phases, this paper further details the algorithmic principles, implementation workflow, and parameter settings of the adaptive error precision adjustment module.

To address the mismatch between a fixed error threshold and the phased operational characteristics during ultrasound scanning, this paper proposes an adaptive error precision adjustment method based on scanning speed variations. In actual clinical operations, physicians typically push the probe at a higher speed during the coarse localization phase to rapidly approach the target region; during this phase, the requirement for trajectory precision is relatively low. Conversely, during the fine adjustment phase, physicians perform delicate adjustments at a lower speed, necessitating higher trajectory precision to ensure the quality of the ultrasound image. Based on these clinical operational characteristics, this paper takes the end-effector’s moving speed in the expert demonstration trajectory as a key indicator reflecting the current operational precision requirement, and constructs a mapping relationship between the scanning speed and the waypoint extraction error threshold, thereby achieving dynamic adaptive adjustment of the error threshold. This process can be formalized as the following constrained optimization problem:

Where $\epsilon_{min}$ and $\epsilon_{max}$ represent the lower and upper limits of the error threshold, respectively, which are used to restrict the variation range of the waypoint extraction precision.

$\nu_{\mathrm{mid}}$ is the critical speed, which controls the central position where the error threshold transitions from large to small.

α is the slope parameter, used to control the sensitivity of the error threshold to speed variations.

When the end-effector’s moving speed is lower than $\nu_{\mathrm{mid}}$, ϵ(ν) gradually approaches $\epsilon_{min}$, and the waypoint extraction precision increases; when the speed is higher than $\nu_{\mathrm{mid}}$ the error threshold is gradually relaxed, thereby reducing the number of waypoints to improve overall efficiency.

(3) Parameter Settings and Algorithm Implementation

To ensure that the parameter settings have a clear basis and are reproducible, this paper conducts a statistical analysis of the demonstration data. Specifically, prior to waypoint extraction, each scanning demonstration recorded during the data acquisition phase is analyzed to calculate the maximum and minimum values of the manipulator’s end-effector moving speed. The average of these two values is taken as the critical speed parameter in the error adjustment function, denoted as ${\;\nu }_{\mathrm{mid}}=3.43\;\mathrm{mm}/s$. Meanwhile, according to the comprehensive requirements of the ultrasound scanning task for efficiency and precision, the upper and lower limits of the error threshold are set to $\epsilon_{max}=0.008$ and $\epsilon_{min}=0.006$ respectively. The slope parameter α is used to control the smoothness of the error threshold’s variation with speed. If the value of α is too large, the error threshold will undergo a nearly step-like change with speed, making the number of waypoints overly sensitive to speed noise; if the value of α is too small, the effect of error adjustment is not obvious, making it difficult to distinguish the precision requirements of different scanning phases. Comprehensively considering the variation range of the end-effector speed during the ultrasound scanning process and the stability of the algorithm, this paper uniformly sets α=1.0 across all experiments to strike a balance between precision adaptability and waypoint extraction stability.

In actual implementation, the end-effector’s moving speed between all time steps in the demonstration trajectory is first calculated, and the corresponding error threshold for each time step is adaptively calculated according to Formula (3). Subsequently, a waypoint extraction algorithm based on a greedy strategy is employed: the starting time step and the ending time step of the demonstration trajectory are fixed as waypoints. Starting from the initial waypoint, the trajectory reconstruction error between the current waypoint and candidate time steps is progressively evaluated. When this error exceeds the minimum value of the error thresholds corresponding to all time steps within this interval, the previous time step is determined as a new waypoint, which serves as a new starting point to continue the aforementioned process until the waypoint extraction for the entire trajectory is completed.

Through the aforementioned adaptive error precision adjustment mechanism based on speed variation, the system can automatically adjust the waypoint extraction precision according to the physician’s operating speed at different scanning stages, achieving a dynamic balance between efficiency and precision in waypoint density. This method effectively overcomes the limitations of fixed error thresholds in complex scanning tasks, providing reliable support for the application of waypoint representation in high-precision, long-duration medical robot tasks such as autonomous ultrasound scanning.

### 2.3. Autonomous Scanning Control Strategy

(1) Smooth End-Effector Pose Control via Velocity Constraints

In waypoint-based predictive control, the spatial distances between adjacent predicted poses can vary significantly. Directly computing joint or end-effector velocities from these pose differentials can induce abrupt velocity spikes. Such spikes not only degrade control precision but also risk triggering overspeed motions (i.e., kinematic limit violations) in the manipulator. Consequently, upon receiving each new waypoint command, this study executes a local online trajectory replanning based on a Trapezoidal Velocity Profile. This mechanism ensures that the end-effector first accelerates or decelerates to an optimal cruising speed, and subsequently approaches the target pose steadily at a constant velocity.

When a new target waypoint $p_{d}$ arrives, let the current position at the start of the segment be $p_{t}$, and the Euclidean distance between the two points is:

(2) $$D=\left\|p_{d}-p_{t}\right\|,$$

The total execution time within the segment is $T\;=\;NT_{S}$, $T_{S}$ is the model prediction cycle, the current cruising speed at the start of each segment is $v_{0}$, and the maximum allowable absolute acceleration is a. Within this segment, acceleration or deceleration is first performed to reach the cruising speed, and the target waypoint $p_{d}$ is reached at the cruising speed. The total displacement within the segment satisfies the following formula:

(3) $$D=\underset{Acceleration\;phase}{\underbrace{\frac{\nu_{0}+\nu }{2}t_{a}}}+\underset{Constant\;velocity\;phase}{\underbrace{\nu \left(T-t_{a}\right)}},t_{a}=\frac{\nu -\nu_{0}}{a}$$

Therefore, the cruising speed for this segment is calculated as

(4) $$v=aT+v_{0}\pm \sqrt{{\left(aT+v_{0}\right)}^{2}-(v_{0}^{2}+2aD)}$$

This achieves an adaptive adjustment of the maximum cruising speed within the segment based on the distance to the waypoint, and the acceleration/deceleration magnitude is subjected to the hard constraints of a and T, ensuring that velocity changes are smooth and controllable.

Unlike the general trajectory control of “automatic deceleration when approaching the target”, the velocity planning in this study directly derives from the operational patterns in the expert demonstration: the physician moves faster during the coarse localization phase, and the waypoints in the trajectory are sparsely distributed; during the fine adjustment phase, the movement slows down, and the waypoint distribution is more dense. Therefore, during the execution phase, the system performs trapezoidal velocity profile planning based on the pose difference between consecutive waypoints: a larger difference indicates that the task is in the fast scanning phase, and the system allocates a higher cruising speed; a smaller difference indicates entry into the fine adjustment phase, and the system lowers the cruising speed to enhance control precision. Consequently, the manipulator can authentically reproduce the physician’s scanning rhythm of “fast localization—low-speed fine adjustment” during operation—without the need for additional manual parameter tuning.

(2) Probe normal constant force maintenance based on force control

To ensure a stable and appropriate contact force between the ultrasound probe and the patient’s skin during the scanning process, an active compliance admittance control strategy is implemented along the probe’s normal vector (*Z*-axis of the tool coordinate system). To preserve the cross-instance universality of the framework, the normal force controller utilizes a simplified second-order dynamic model, directly mapping contact force errors to normal compensation velocities within the task space. The dynamic equilibrium equation is expressed as:

(5) $$M\;{\overset{..}{x}}_{normal}+D\;{\dot{x}}_{normal}=\Delta F$$

where M and D denote the desired inertia and damping parameters along the normal direction within the task space, respectively; ${\overset{..}{x}}_{normal}$ and ${\dot{x}}_{normal}$ represent the normal compensation acceleration and velocity, respectively (the system integrates ${\overset{..}{x}}_{normal}$ at each control cycle to yield the final commanded normal compensation velocity, denoted as $v_{normal}$). Furthermore, ΔF represents the contact force error of the ultrasound probe, defined as follows:

(6) $$\Delta F=F_{e}-F_{d}$$

where $F_{e}$ denotes the actual contact force of the probe measured by the force sensor, and $F_{d}={\left\{0,0,f_{z},0,0,0\right\}}^{T}$ signifies the desired force specified across various probe axes. Given that this study exclusively executes force control along the probe’s *Z*-axis, we define $f_{z}=12\;N$. This magnitude sits at the median of the typical 10–15 N pressure range applied during clinical expert manipulation. It achieves an optimal balance between ensuring high-quality acoustic windows and preventing excessive tissue compression. By achieving an orthogonal decoupled fusion of the velocity smoothing control strategy and the constant normal force control strategy within the task space, the system realizes safe, stable, and fine-grained autonomous kidney ultrasound scanning across different manipulator instances with objective physical parameter deviations.

(3) Decoupled Fusion of Motion and Force alongside Safety Redundancy Mechanisms

To address the complex interaction requirements between motion trajectories and contact forces during clinical scanning, this study establishes an orthogonal decoupled fusion control architecture within the task space and designs multi-level redundant protection mechanisms to guarantee scanning safety.

#### 2.3.1. Orthogonal Decoupling and Command Fusion in the Task Space

The system establishes a local tool coordinate frame at the probe’s end-effector, decoupling the scanning task within the spatial dimension: the trapezoidal velocity planning algorithm operates exclusively on the tangent plane where the probe slides along the body surface, generating smooth tangential velocity components between waypoints. Conversely, the constant-force admittance controller is strictly confined to the normal axis perpendicular to the body surface, calculating the normal compensation velocity based on real-time force errors.

During each real-time low-level control cycle, the system performs an orthogonal vector synthesis of the tangential velocity planning component $v_{tangent}$ and the normal force compensation velocity component $v_{normal}$:

(7) $$v_{total}=v_{tangent}+v_{normal}$$

This orthogonal decoupling strategy mathematically circumvents control conflicts between position servoing and force servoing on the same degree of freedom. It ensures that the probe, while scanning smoothly, can independently and compliantly adapt to normal displacements induced by human respiration. Furthermore, the synthesized total target velocity in the task space, $v_{total}$, is fed directly into the Inverse Kinematics (IK) solver of the target manipulator, where it is converted into low-level joint velocity commands. This mechanism completely isolates the high-level task planning from interference caused by specific equipment base calibration and D-H parameter discrepancies, providing a robust foundational guarantee for the system’s cross-instance reusability.

#### 2.3.2. Triple Safety Redundancy Mechanisms for Policy Latency

Given the stringent requirements for physical interaction safety in medical scenarios, coupled with the inherent inference latency (approximately 70 ms) stemming from the massive parameter scale of the high-level policy model (ACT model), the system’s constant-force control, inverse kinematics (IK) resolution, and safety monitoring loops operate entirely independently within the manipulator’s low-level real-time control kernel. Their closed-loop control cycle is strictly regulated at the 10 ms level. Even if the policy model experiences command update lags in highly dynamic scenarios, the low-level system can still perform real-time fine-tuning of contact forces and motion poses at a high-frequency cycle far exceeding human reaction speeds. This not only fundamentally isolates potential physical safety risks triggered by high-level algorithm latency but also effectively guarantees fine-grained trajectory tracking precision between adjacent waypoints at the foundational level. Additionally, the system introduces a triple protection mechanism, comprising:

(1) Software-Level Velocity Saturation: Following vector synthesis, both the tangential trapezoidal velocity planning output and the normal admittance control compensation velocity undergo strict kinematic saturation before being dispatched to the joint controllers. This ensures that velocities never exceed predefined maximum thresholds, fundamentally preventing the manipulator from overspeeding or losing control due to abnormal high-level model outputs or abrupt trajectory mutations.
(2) Real-Time 6-DOF Force Monitoring and Zero-Force Compliant Release: The low-level system monitors the six-dimensional force/torque data at high frequencies in real time. Upon detecting that the contact force or torque in any dimension exceeds predefined physical safety hard limits, the system forcefully interrupts the current trajectory tracking task and instantaneously switches the manipulator into a zero-force compliant dragging mode. In this highly compliant state, the probe yields to external forces; clinicians can swiftly detach the probe from the human body with a light, single-handed push, thereby maximizing the subject’s protection against crush injuries.
(3) Real-Time Supervision and Emergency Stop: As the ultimate safety baseline, an independent physical emergency stop (E-stop) button is equipped at the hardware level. Throughout the entire autonomous scanning process, experienced professional operators are stationed nearby for real-time monitoring. Should they anticipate severe subject discomfort or detect abnormal system poses, operators can immediately depress the E-stop button, instantaneously cutting off the low-level servo power supply to ensure absolute physical safety.

### 2.4. Experimental Setup and Evaluation Metrics

To comprehensively validate the effectiveness of the proposed method and its zero-shot deployment capability across different equipment instances with objective physical parameter deviations, this study designed an autonomous scanning experiment targeting the human kidney long-axis standard plane. The experimental protocol comprises two phases: initially, model training is executed on the source-domain manipulator (i.e., the data acquisition platform); subsequently, the trained model is deployed and evaluated on both the source-domain manipulator and the target-domain manipulator (i.e., the skill reproduction platform) to rigorously assess its kinematic-level cross-instance generalization performance.

#### 2.4.1. Experimental Setup and Comparison Baselines

(1) Setup of the Experimental Platform with Physical Discrepancies

**Figure 6:**
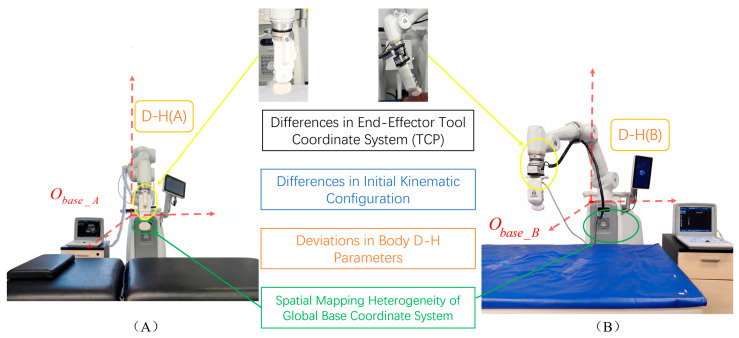
Experimental Platform (**A**) Source Domain Manipulator (**B**) Target Domain Manipulator.

The hardware environment is illustrated in Figure 6. Although both the data acquisition and skill reproduction platforms utilize the same HEBIN-RUS-T7 collaborative manipulator series, to simulate objective physical deviations in real-world clinical deployment and construct a rigorous testing environment, the two systems exhibit significant inconsistencies across the following four dimensions in their low-level configurations.

1. **Tool Offset Discrepancies** (highlighted in yellow at the top of Figure 6): The source-domain manipulator employs a standard probe holder, whereas the target domain utilizes a custom 3D-printed fixture. Consequently, their Tool Center Point (TCP) offset matrices are entirely distinct.
2. **Initial Kinematic Configuration Discrepancies** (highlighted in blue in the middle of Figure 6): The two manipulators are initialized with different scanning poses. This implies that even when targeting the identical task-space objective, their Inverse Kinematics (IK) generated joint motion trajectories are completely different. This effectively eliminates the risk of the model degrading into merely memorizing the joint state features of a single equipment instance.
3. **Physical D-H Parameter Deviations** (highlighted in orange in the middle of Figure 6): Constrained by manufacturing and assembly tolerances, as well as mechanical wear from long-term service, the actual link lengths and joint offsets of the two manipulators are inconsistent. This leads to fundamental divergences in their low-level kinematic models.
4. **Base Frame Spatial Mapping Discrepancies** (highlighted in green at the bottom of Figure 6): Due to different manipulator mounting positions, massive translational and rotational deviations exist between the base coordinate frames of the source and target instance relative to the subject.

To quantify these differences, Table 1 details the core physical kinematic offsets of the target domain relative to the source-domain instance. In summary, it is precisely because of these four categories of objective physical discrepancies—spanning from low-level kinematic deviations to macroscopic deployment shifts—that the inverse kinematics models of the two systems are completely incompatible. Under this “physical parameter barrier,” any traditional policy relying on joint-space representations will inevitably fail. This provides a rigorous and realistic testbed to verify that the waypoint representation method can effectively decouple low-level physical parameters. Furthermore, both systems are equipped with the identical Sonostar SS-6 ultrasound machine and host computer to ensure consistency in the imaging and computational environments.

(2) Baseline Methods and 2 × 2 Cross-Comparative Design

To rigorously evaluate the superiority of the proposed waypoint representation strategy in executing long-horizon fine-grained manipulation tasks concerning temporal error suppression, alongside its cross-architecture universality (i.e., plug-and-play capability) as a task-space representation, this study transcends the constraints of a single backbone network and designs a 2 × 2 cross-comparative experiment.

First, regarding the selection of the foundational backbone network, in addition to the Action Chunking Transformer (ACT) [15] employed by our complete framework, the experiment specifically introduces the current state-of-the-art Diffusion Policy (DP) [17] from the imitation learning domain as an external baseline.

Second, concerning the action space representation, the following two strategies are configured for the aforementioned backbone networks to formulate cross-combinations:

1. Incremental Representation (Traditional Baseline): This adopts a strategy of “predicting the continuous pose increment for the subsequent timestep based on the current state”. While this approach reflects the mainstream paradigm for executing continuous tasks—as exemplified by prior works such as [9]—its intrinsic reliance on high-frequency relative positional updates renders it highly susceptible to severe error accumulation during long-horizon tasks.
2. Waypoint Representation (Proposed Herein): The model directly predicts a sequence of key waypoints endowed with global reference coordinates within the task space. The waypoints extracted via our adaptive algorithm can learn trajectory distributions possessing global geometric constraints. This aims to utilize sparse yet critical absolute pose points to guide manipulator motion, thereby mechanistically mitigating error drift in long sequences.

Through these configurations, the experiment establishes four comparative models: ACT-Incremental, ACT-Waypoint, DP-Incremental, and DP-Waypoint.

Safety and Evaluation Declaration: Given the stringent physical safety requirements inherent to in vivo medical robotic ultrasound interactions, it is imperative to absolutely circumvent the risks of uncontrollable trajectory divergence and potential crush injuries that exploratory baseline models (e.g., the raw incremental model) might trigger in complex dynamic environments. Consequently, the aforementioned cross-validation and quantitative comparisons regarding network architectures and action representations are strictly conducted on offline datasets. Only the complete proposed framework (ACT-Waypoint)—which is safeguarded by the high-frequency 10 ms low-level admittance force control and triple safety redundancies—is approved for subsequent real-world in vivo human scanning validation. This ensures absolute physical safety during the cross-instance deployment process.

#### 2.4.2. Evaluation Metrics

To concurrently ensure the clinical usability of the medical images and the experimental reproducibility of the robotic task, this study establishes a comprehensive evaluation framework encompassing two dimensions: qualitative expert assessment and quantitative geometric image evaluation.

(1) Qualitative Metric (Expert Subjective Assessment)

**Figure 7:**
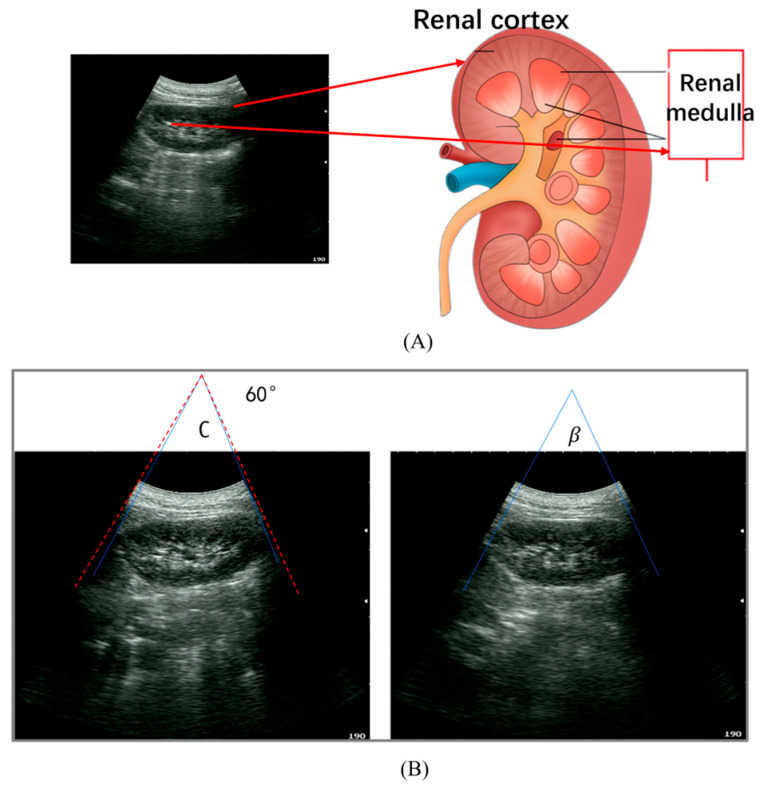
Evaluation Metrics: (**A**) Schematic of the ultrasound image structure of the renal long-axis standard plane; (**B**) Schematic of the proportion of the long-axis standard plane.

To guarantee that the acquired ultrasound images satisfy the stringent diagnostic requirements of clinical practice, this study invited two senior sonographers with extensive clinical experience (>5 years) to conduct an independent blinded evaluation of all scanning results. A successful, clinically viable autonomous scan requires a consensus between both sonographers and must simultaneously satisfy the following two critical criteria:

1. Anatomical Structural Integrity: The ultrasound image must distinctly visualize the complete contour of the renal cortex and clearly resolve the renal medulla [18] (as illustrated in Figure 7A). This satisfies the sonographers’ fundamental acoustic window requirements for lesion localization.
2. Standard Plane Accuracy: The imaging plane must precisely align with the maximum coronal plane of the kidney’s long axis, devoid of significant deviations or off-axis truncations. This ensures the precision of subsequent morphological parameter measurements (e.g., maximum renal length).

(2) Quantitative Geometric Metric

Building upon the expert evaluation, an objective metric derived from image geometric information is introduced to quantify the degree of task completion. Given that the kidney long-axis standard plane occupies a specific angular range within the sector-shaped ultrasound Field of View (FoV), the degree of task completion is defined as the ratio of the subtended angle of the renal anatomical structure to the total imaging FoV angle of the probe:

(8) $$P=\frac{\beta }{C}$$

where *β* denotes the subtended angle corresponding to the identified kidney long-axis standard plane in the ultrasound image at the termination of the scan; *C* represents the physical imaging Field of View (FoV) angle of the ultrasound probe (as illustrated in Figure 7B). Based on the statistical analysis of extensive clinical data and prior expert knowledge, this study establishes *p* ≥ 0.85 as the quantitative threshold for task success. Specifically, the autonomous scanning task is deemed successful when the renal structure occupies more than 85% of the image; otherwise, it is considered a failure. The ultimate success rate reported in this study synthesizes both subjective and objective metrics; a trial is counted as a successful validation of cross-instance skill transfer only when both criteria are simultaneously fulfilled.

## 3. Results

### 3.1. Analysis of Model Prediction Accuracy

To comprehensively and rigorously evaluate, from a statistical perspective, the reproduction capability of the waypoint representation strategy concerning expert demonstration trajectories, alongside its cross-model universality—thereby providing robust theoretical and empirical support for subsequent physical deployment—this study conducted a 2 × 2 cross-comparative experiment on the offline validation set. This involved introducing the state-of-the-art Diffusion Policy (DP) as an external architectural baseline to compare against the Action Chunking Transformer (ACT). Each model architecture configuration underwent 25 independent repeated trials. By calculating the Euclidean distance between the predicted trajectories and the original expert demonstration paths, the system aggregated the Mean Error, Standard Deviation (SD), and Maximum Error across the various combinations of network architectures and representation strategies. Furthermore, to facilitate formal statistical analysis, this study computed the 95% Confidence Interval (CI) for the mean errors and executed independent samples *t*-tests to evaluate the different representation strategies (Incremental vs. Waypoint) within identical architectures. The results are presented in Table 2.

The empirical results demonstrate that, regardless of whether the underlying backbone network employs ACT or Diffusion Policy, the proposed waypoint representation exhibits highly significant statistical superiority in terms of trajectory reproduction precision and stability. For the ACT architecture, the mean error of the waypoint model (ACT-Waypoint) is 3.3 mm, representing a reduction of approximately 70.5% compared to the incremental baseline (11.2 mm). Similarly, even within the highly generative Diffusion Policy (DP) architecture, the traditional incremental action representation continues to exhibit a high mean error (9.8 mm) and severe maximum error extremes (12.5 mm) due to long-horizon error drift. Upon introducing the waypoint strategy, the mean error for the DP architecture also plummets to 3.1mm.

Independent samples t-test results indicate that the precision improvements yielded by the waypoint strategy across both architectures possess extremely high statistical significance (p<0.001), effectively precluding the confounding influence of random errors. Furthermore, evaluating the dispersion of the error distributions, both model variants employing the waypoint representation constrain their error Standard Deviations (SD) to a low level of approximately 1.5 mm. Their 95% Confidence Intervals (CI) for the mean errors are exceptionally narrow; not only are their upper bounds substantially lower than the lower bounds of the corresponding incremental models, but their maximum errors also reside securely within the clinically acceptable safety tolerance range.

This 2 × 2 cross-comparative experiment conclusively demonstrates, from both statistical and engineering perspectives, that by directly predicting key waypoints within the task space, the model mathematically and mechanistically thwarts the error accumulation effect inherent in long-horizon tasks. This strategy not only drastically enhances absolute predictive precision but also manifests exceptional cross-generative architectural universality (i.e., plug-and-play capability). Given that the ACT-Waypoint combination aligns more synergistically with the low-latency inference potential requisite for real-time force control feedback, this study will exclusively employ this configuration as the core deployment system in the subsequent physical scanning validation during cross-instance deployment.

### 3.2. Performance Evaluation of Cross-Physical Instance Zero-Shot Deployment

To further evaluate the policy’s zero-shot deployment performance in a real-world dynamic environment, this study selects the ACT-Waypoint model as the core execution scheme, leveraging its representation advantages demonstrated in offline experiments. This system integrates millisecond-scale low-level orthogonal decoupled force control in the task space alongside triple safety redundancies. Compared to high-latency end-to-end models, this system is better equipped to compensate for dynamic deviations induced by respiratory perturbations. On two equipment instances with incompatible low-level kinematic parameters, the system successfully completed 25 independent in vivo autonomous kidney scanning tasks.

**Figure 8:**
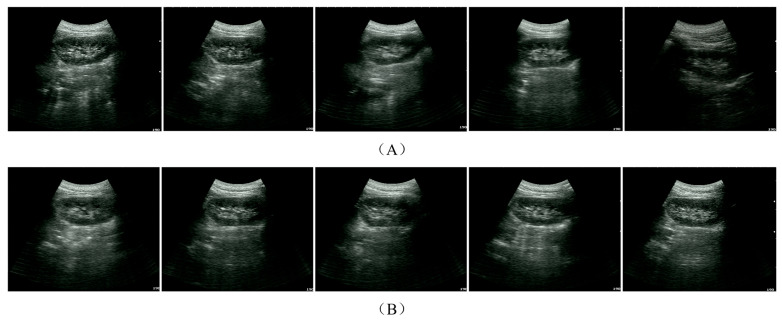
(**A**) Experimental Results of Autonomous Scanning for the Source Domain Manipulator. (**B**) Experimental Results of Autonomous Scanning for the Target Domain Manipulator.

To ensure that the evaluation results possess authentic clinical reference value, the criteria for task success incorporate two stringent standards: qualitative expert assessment and quantitative image geometric evaluation. Two senior sonographers with extensive clinical experience (>5 years) were invited to conduct an independent blinded evaluation of all experimental images. A single scan is recorded as successful only when the final ultrasound plane simultaneously satisfies: (1) Qualitative expert consensus (both sonographers agree that the image exhibits no off-axis deviations or truncations, with clearly identifiable renal cortex and renal medulla); and (2) Quantitative task completion degree p≥0.85. Based on these rigorous diagnostic-grade standards, the task success rates of various models were statistically analyzed across both the source-domain (data acquisition) instance and the target-domain (skill reproduction) instance. The results are summarized in Table 3 (representative experimental results are shown in Figure 8).

The empirical results demonstrate that the proposed waypoint-based model exhibits remarkable cross-instance generalization capabilities. Deployed on the source-domain manipulator, the model achieved a high task success rate of 92% (95% CI: [75.0%, 97.8%]). Crucially, on the target-domain equipment—which was entirely excluded from the training phase and inherently possesses an objective physical parameter barrier—the model robustly maintained an 84% success rate (95% CI: [65.3%, 93.6%]). In stark contrast, the incremental model acting as the traditional baseline (predicated on Cartesian pose increment predictions) exhibited conspicuous performance degradation, yielding success rates of merely 76% and 64% on the respective manipulator instances.

An in-depth data analysis reveals that the performance drop of the waypoint model on the target domain (8%) is not only substantially smaller than that of the incremental model (12%), but statistical analysis utilizing the 95% Confidence Intervals uncovers a more profound finding: the lower bound of the waypoint model’s CI on the target domain (65.3%) still surpasses the mean performance of the incremental model on the target domain (64%). This highly significant statistical superiority conclusively verifies that the waypoint representation demonstrates enhanced robustness against low-level equipment parameter shifts and assembly tolerances. It fundamentally overcomes the severe risks of policy failure inherent in traditional incremental strategies when confronted with physical deployment discrepancies.

Failure Case Analysis: To further evaluate the performance boundaries of the system, this study analyzed the 6 unsuccessful cases out of the 50 in vivo scanning experiments (as shown in Table 3). Among these, one failure occurred during the initial positioning phase, where the probe failed to identify the renal long-axis standard plane. The remaining five failures occurred during the micro-posture adjustment phase; although the system successfully navigated to the vicinity of the target region, the final imaging plane failed to meet the geometric metric of p≥0.85, or exhibited insufficient anatomical resolution of the renal cortex and medulla. Analysis indicates that these insufficiencies in fine-tuning precision can be partially attributed to the limitations of the “single volunteer” sample: since the training data is derived solely from the 180 trajectories of a single subject, the ultrasound image feature distribution learned by the model is relatively narrow. Consequently, during actual scanning, the system’s generalization and fine-tuning capabilities are somewhat constrained when confronted with local dynamic image perturbations caused by respiratory motion, slight deviations in probe contact angles, or soft tissue deformation under pressure.

### 3.3. Stability of Contact Force Control

**Figure 9:**
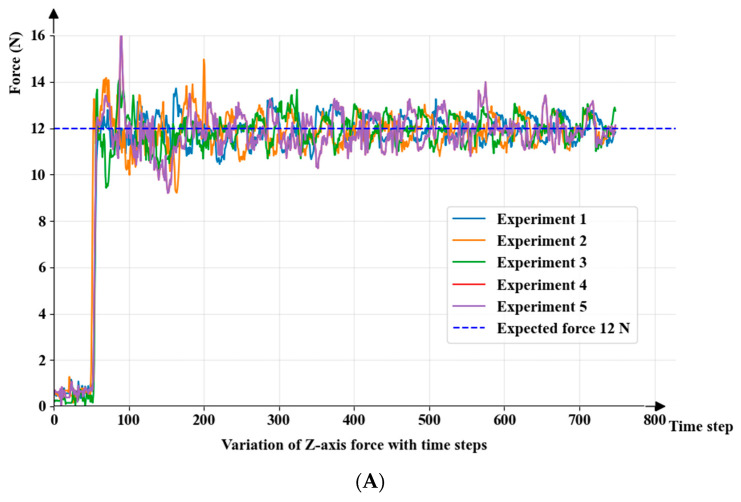

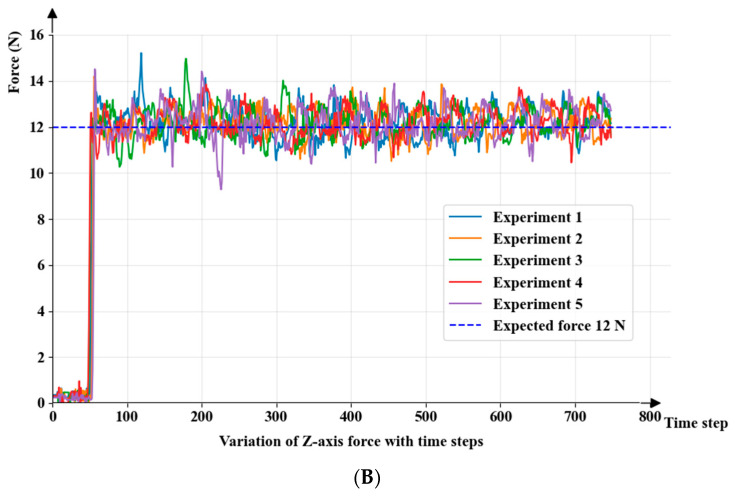
Experimental Results of Force Control Accuracy: (**A**) *Z*-axis Force Control Response Curve of the Source Domain Manipulator; (**B**) *Z*-axis Force Control Response Curve of the Target Domain Manipulator.

To validate the control precision and robustness of the proposed method regarding the end-effector contact force during cross-instance deployment tasks, this study conducted 5 independent autonomous scanning force control experiments on both the source-domain (data acquisition) manipulator and the target-domain (skill reproduction) manipulator, respectively. The desired normal contact force of the probe was set to $F_{d}\;=\;12\;N.$ Free-space motion data recorded prior to skin contact were excluded to specifically focus the statistical analysis on the mechanical performance during the steady-state phase following contact establishment. The force control response curves for both manipulators are illustrated in Figure 9.

(1) Analysis of the Dynamic Response Process

As can be seen from the time-domain curves in Figure 9, both manipulator instances exhibited highly consistent force control characteristics. During the contact establishment phase (Contact Establishment Phase), both systems were able to rapidly converge to the vicinity of the expected force without overshoot within approximately 1.4 s (about 10 time steps). After entering the steady scanning phase (Steady Phase), although the subject’s respiratory motion caused periodic non-rigid displacement of the chest and abdomen (leading to passive contact force fluctuations), the PI force control algorithm was able to achieve rapid active compensation, consistently maintaining the force value within a dynamic equilibrium band centered at 12 N, without divergence or severe oscillation.

(2) Quantitative Error Statistical Comparison

To further evaluate the control discrepancies prior to and following cross-instance deployment, Table 4 summarizes the statistical results of the steady-state data post-contact:

The comparative data indicates that the system did not exhibit any conspicuous performance degradation upon deployment to the target-domain instance. Although the mean force value of the target-domain manipulator experienced a marginal offset (+0.19 N, primarily attributed to minor residual errors during sensor initialization and end-effector payload gravity calibration, which falls well within the system’s allowable tolerance), its force control standard deviation (0.69 N) remained on par with that of the source domain (0.72 N), and was even marginally optimized. This corroborates that the orthogonal decoupled admittance force control strategy of the proposed system possesses exceptionally strong cross-instance robustness. It can consistently guarantee compliant and stable contact between the probe and human tissue in the presence of objective physical assembly and parameter deviations, providing critical support for acquiring high-quality ultrasound images and ensuring absolute patient safety.

### 3.4. Ablation Study of the Adaptive Strategy

To comprehensively evaluate the effectiveness of the various system components and parameter configurations proposed in this study, and to delineate the independent contributions of distinct modules to the cross-physical instance deployment performance, this section conducts an in-depth ablation analysis across two hierarchical levels: “Core System Modules” and “Adaptive Precision Regulation”.

#### 3.4.1. Core System Module Ablation

We initially constructed four progressive system variants for comparative validation. As delineated in Table 5, this subset of experiments rigorously evaluates the independent contributions of the action representation modalities (Incremental vs. Waypoint) and the low-level physical control strategies (Pure Position Servoing vs. Task-Space Hybrid Force Control).

Analysis and Discussion of Module Contributions:

Superiority of Waypoint Representation (Combating Error Accumulation): Comparing Version 1 and Version 2 reveals that merely switching the control space from “continuous Cartesian increment prediction” to “fixed-precision waypoint prediction” drastically reduces the system error from 11.2 mm to 4.5 mm. This demonstrates that the waypoint mechanism, by sparsifying temporal predictions, mathematically and mechanistically thwarts the error accumulation effect inherent when executing long-horizon imitation learning across equipment with underlying physical deviations.

Physical Necessity of Low-Level Force Control (Phantom-Based Validation): It is imperative to explicitly declare that, out of strict consideration for medical ethics and the absolute safety of human subjects, “Version 3”—which strips away the trapezoidal velocity planning and normal admittance control—could not be validated in an in vivo human environment. Instead, we conducted ablation testing on this version using a dynamic abdominal phantom equipped with simulated respiratory perturbations. The results indicate that when the waypoints predicted by the high-level policy model are directly dispatched to the robot for pure position servoing, the system is entirely incapable of adapting to dynamic soft tissue displacements. During testing, this version frequently exhibited probe detachment from the acoustic window (with the contact force plummeting to 0) or generated excessively high local pressure exceeding safety thresholds, thereby triggering the system’s emergency stop protection. This conclusively corroborates that low-level physical force control is not merely an “optional optimization” for improving accuracy by a few millimeters, but rather a “mandatory prerequisite” for the safe deployment of imitation learning models in real-world dynamic clinical environments.

#### 3.4.2. Adaptive Threshold Parameter Ablation

Having established the structural advantages of adaptive waypoints and force control, this study further conducts an ablation on the core parameters of the “velocity-based adaptive error mapping function”. This mechanism aims to emulate the clinician’s manipulation techniques by relaxing the threshold during the coarse scanning phase to enhance efficiency, and tightening the threshold during the fine-tuning phase to guarantee precision. We compared multiple sets of fixed thresholds and adaptive parameter combinations on the validation set, with the results presented in Table 6.

Parameter Analysis and Discussion: In conjunction with Table 4, it is evident that the adaptive error regulation strategy significantly outperforms fixed thresholds in breaking the “zero-sum trade-off between precision and efficiency”. Taking the fixed threshold ϵ=0.0015 as an example, although it achieved a passable error performance (4.5 mm), the mandatory high-frequency waypoint extraction throughout the entire process resulted in an excessively lengthy action sequence (670 steps), making it highly susceptible to inducing high-frequency execution jitter on the physical platform. Conversely, by introducing the adaptive mapping function, the model can significantly optimize the step count while concurrently ensuring dynamic tracking precision.

When adopting the adaptive combination of $\epsilon_{max}=0.004$ and $\epsilon_{min}=0.002$ the model achieves a precision of 4.2 mm and an 88% success rate within a relatively short duration (460 steps). In routine clinical scanning on a single source-domain instance, considering the clinicians’ requirements for scanning efficiency, this parameter setting could be regarded as the comprehensive optimal configuration. However, the core challenge and application scenario of this study lie in “cross-instance zero-shot deployment in the face of physical assembly and parameter deviations”. When confronting the highly uncertain low-level physical parameter shifts and unseen dynamic perturbations of the target domain, the system’s fault tolerance is exceptionally low; therefore, the priority of the subject’s physical interaction safety and trajectory precision must absolutely supersede the optimization of execution time.

Consequently, in the final in vivo cross-physical instance deployment, this system deliberately adopted a more stringent parameter configuration of $\epsilon_{max}=0.002$ and $\epsilon_{min}=0.001$. Empirical data corroborates that, compared to the aforementioned comprehensive configuration, this conservative setup limits the increase in scanning time to an acceptable margin (510 steps, an increase of only about 10%) while drastically reducing the average trajectory error to 3.3 mm and achieving the highest task success rate of 92%. This decision perfectly aligns with the stringent demands for “ultimate safety and precision” when tackling underlying physical parameter barriers.

## 4. Discussion

### 4.1. Mechanisms of Cross-Instance Deployment and Error Accumulation Suppression

To address the critical bottleneck pervasive in the medical robotics domain—namely, the difficulty in reusing expert demonstration data and adapting to parameter shifts in actual clinical equipment—this study proposes a waypoint-based imitation learning framework. The empirical results verify that this framework not only achieves a 92% scanning success rate on the source-domain manipulator but also maintains a high performance of 84% on the target-domain equipment instance. This target instance was completely excluded from training and inherently possesses low-level parameter barriers, thereby demonstrating exceptional zero-shot deployment capabilities.

Compared to the traditional incremental strategy (Cartesian Increment), the waypoint-based strategy adopted herein exhibits significant advantages in cross-instance reusability by overcoming physical parameter deviations. Its underlying mechanisms can be attributed to the following two aspects:

1. Task-Space Absolute Expression (vs. Joint Space Strategies): Distinct from methods that achieve skill reproduction by imitating joint angles, the “waypoints” extracted in this study are absolutely defined within the task space. This representation thoroughly decouples the “clinician’s intent” (i.e., the absolute pose of the probe on the anatomical structure) from the “low-level kinematic parameters” (i.e., how to resolve the link motions to reach that pose). Consequently, even if the target-domain manipulator instances possess significantly different base deployment locations and physical D-H parameters (as shown in Figure 6), the model can still accurately transfer the global scanning intent. This is the prerequisite for achieving reliable cross-instance deployment.

2. Effective Suppression of Long-Horizon Error Accumulation (vs. Incremental Baseline Strategy): Ultrasound scanning is inherently a long-horizon task. The incremental model relies on dense timestep predictions, implying that completing a full scan requires executing thousands of continuous incremental inferences. During this process, any minute prediction error or execution deviation in a single step—especially during long-term clinical service, where internal joint wear and frictional resistance lead to severe degradation in low-level positioning accuracy—will be retained and propagated to the subsequent step. This generates the classic error accumulation problem, causing the trajectory deviation to amplify quadratically over timesteps.

Conversely, the waypoint-based strategy employed in this study is fundamentally a mechanism of temporal abstraction. By predicting sparse key waypoints, the model drastically truncates the originally lengthy decision sequence. This macro-step prediction strategy significantly curtails the decision frequency, thereby severing the propagation chain of error accumulation at its source. As shown in Table 2, the waypoint model not only yields an exceptionally low mean error (3.3 mm) but also a standard deviation (1.4 mm) far smaller than that of the incremental model (4.2 mm). This fully substantiates the absolute superiority of this strategy in maintaining global trajectory consistency and suppressing error accumulation during long-horizon tasks.

### 4.2. Clinical Biomimetic Significance of the Speed-Accuracy Adaptive Strategy

Beyond the zero-shot deployment capability across physical instances, another core finding of this study is the critical role of the “velocity-based adaptive error regulation mechanism” in enhancing the efficiency and **biomimetic naturalness** of autonomous scanning. The ablation study results in Table 3 indicate that when adopting the optimal adaptive parameter combination ($\epsilon_{max}=0.004,\epsilon_{min}=0.002$), the model, while maintaining a high success rate (88%), reduced timestep consumption by approximately 31% (460 vs. 670) compared to the high-precision fixed-threshold strategy. The **fundamental** reason for this performance enhancement is that the algorithm successfully emulates the implicit “Speed-Accuracy Trade-off” cognitive strategy employed by sonographers during clinical manipulation:

Rapid Approximation during the Coarse Positioning Phase: In the initial stage of locating the renal acoustic window, clinicians typically move the probe swiftly to cover a larger search area. At this stage, although the motion velocity is high, the requirements for absolute trajectory precision are relatively relaxed. By perceiving the high-speed state of the end-effector, the adaptive algorithm automatically relaxes the error threshold, thereby generating a sparse sequence of waypoints. This not only alleviates the computational burden on the model but also enables the manipulator to approach the target region rapidly with smoother and more “confident” motions. It fundamentally circumvents the motion lag caused by oversampling in non-critical regions, a common flaw in traditional fixed-threshold methods.

Dense Regulation during the Fine Scanning Phase: As the probe approaches the target anatomical structure or requires fine-tuning of the imaging plane angle, the clinician significantly decelerates the motion to acquire a clear and stable image. Correspondingly, the algorithm automatically tightens the error threshold to generate a high-density sequence of waypoints, compelling the manipulator to execute high-precision micro-displacements. This dynamic regulation mechanism ensures that during the critical imaging phase, the model can meticulously capture the most subtle manipulative adjustments inherent in the expert demonstration.

In summary, adaptive waypoint extraction is not merely a data compression technique; more fundamentally, it endows the manipulator with an operational “rhythm” akin to that of a human expert. This biomimetic characteristic enables the autonomous scanning system to adapt more naturally to the dynamic interaction requirements across different scanning phases, significantly elevating examination efficiency while guaranteeing diagnostic-grade imaging quality.

### 4.3. Performance Comparison Analysis with Existing Advanced Methods

To objectively position the contributions of this study within the field of autonomous medical robotics, Table 7 benchmarks the proposed waypoint-based model against representative autonomous scanning systems developed for various internal organs (spine, heart, and kidney) in recent years.

The data indicates that in the highly challenging in vivo kidney scanning task, the proposed method achieved a mean position error of 3.3 mm and a task success rate of 92%. Its comprehensive performance is significantly superior to the spine lumbar scanning model in [19] (error: 7.90 ± 6.87 mm, success rate: 79.0%) and the heart scanning model in [20] (success rate: 72%). This performance enhancement can be primarily attributed to the following two technical breakthroughs:

Active compensation for soft tissue dynamic characteristics: Unlike rigid skeletal structures such as the spine, the kidney, as a soft tissue organ in the abdominal cavity, is significantly affected by respiratory motion (displacement amplitude can reach 10–40 mm [22]). The methods in references [19,21] mostly focus on the visual servoing of static anatomical structures and lack active adjustment of dynamic contact force. In particular, although ref. [21] achieved a high success rate (96.7%) in phantom experiments, because the phantom is a static rigid body and lacks the complex respiratory motion and tissue deformation characteristics in a real human body, its method is difficult to generalize directly to a clinical in vivo environment. By contrast, our study maintained a high success rate of 92% in genuine in vivo experiments subjected to human respiratory perturbations and successfully achieved zero-shot deployment across physical instances. This is primarily attributable to the admittance model-based constant normal force control strategy in the task space introduced in this study (as detailed in Section 2.3). This strategy enables real-time compliant compensation for respiration-induced tissue deformation, effectively maintaining a stable physical coupling between the probe and the body surface. This drastically reduces the image loss rate caused by contact instability.

Global optimization capability of the end-to-end strategy: Ref. [20] adopts a hierarchical control architecture (visual recognition + independent motion planning), where cascading errors between independent modules are prone to trigger severe error accumulation. In contrast, the Transformer-based end-to-end model for waypoint prediction proposed herein can directly map from the original ultrasound images to the optimal trajectory in the task space. By utilizing attention mechanisms to capture long-term dependencies, the model exhibits higher precision and robustness in global path planning.

In summary, the proposed method demonstrates explicit technological advantages in handling respiration-perturbed, highly dynamic soft tissue scanning tasks. It not only overcomes the deployment conundrum where low-level physical parameter shifts across different equipment instances render expert demonstration data difficult to reliably reuse, but it also provides robust theoretical and practical engineering support for realizing clinical-grade, high-precision, and highly safe autonomous ultrasound diagnostics.

### 4.4. Research Limitations and Future Challenges

Synthesizing the aforementioned experimental results and the in-depth analysis of the failed scanning cases, although this study has preliminarily verified the feasibility of the physical-parameter-decoupled control architecture in cross-instance deployment, it still faces a series of core bottlenecks before achieving robust application in full clinical scenarios. Measured against the stringent standards of clinical scanning, the current system still exhibits the following limitations in terms of subject coverage, system real-time performance, the breadth of cross-instance universality, and the temporal stability of actions, which urgently require resolution in subsequent work:

(1) Homogeneous Clinical Subject Sample and Generalization Limitations: The current physical validation of this study primarily focuses on verifying the low-level parameter-decoupled control architecture of the robot and achieving a proof-of-concept. Therefore, the dataset utilized for experimental evaluation comprises 180 scanning trajectories acquired from a single healthy subject (a 24-year-old male). Although this dataset yielded effective results in validating the system’s ability to overcome low-level physical parameter deviations and ensure interaction safety, constrained by the sample size, the current results are insufficient to fully substantiate the system’s acoustic robustness and broad clinical generalization capabilities when confronted with drastic variations in ultrasound image appearances. In a genuine clinical environment, discrepancies in age, gender, and BMI among different subjects, alongside pathological structural distortions within the kidney (e.g., cysts, calculi), will all induce significant shifts in feature distributions. Future research will be dedicated to introducing a diversified, clinically representative subject cohort through multicenter clinical trials, aiming to comprehensively evaluate and enhance the model’s generalization performance under complex human anatomical structures.
(2) Constraints of System Computational Latency in Extreme Dynamic Scenarios: Although the system’s low-level millisecond-scale normal admittance force control effectively guarantees physical interaction safety, the massive parameter count of the end-to-end model introduces approximately 70 ms of computation and transmission latency in high-level visual inference and trajectory planning. When confronted with sudden large-scale bodily movements or severe coughing by the patient due to pain, this lag in high-level vision and decision-making may cause the probe to instantaneously slip out of the optimal acoustic window, triggering an interruption of the scanning task. Future work intends to introduce lightweight networks to accelerate inference or integrate a Kalman Filter for state prediction during visual tracking loss, thereby further mitigating the impact of latency on the success rate in extreme dynamic scenarios.
(3) Limitations in the Dimensionality of Equipment Kinematic Variations: Constrained by current laboratory equipment conditions, the zero-shot cross-physical instance deployment in this study was primarily validated between two manipulators of the same series (HEBIN-RUS-T7). It must be emphasized that despite their similar physical appearances, the artificial configuration of different base coordinate mappings, coupled with authentic physical D-H parameter deviations, rendered the Inverse Kinematics (IK) solvers of the two systems entirely incompatible. This has rigorously proven, from a mathematical essence, that the waypoint representation method can effectively decouple low-level physical parameters to realize reliable cross-physical instance deployment. Nevertheless, this still fails to fully encompass the complexities of “truly cross-brand” systems. Future research will focus on deploying this framework onto industrial or medical manipulators with diverse kinematic configurations (e.g., 6-DOF vs. 7-DOF), distinct rated payloads, and disparate proprietary low-level control interfaces, to comprehensively verify its universality within a broad clinical equipment ecosystem.
(4) Temporal Dependency Limitations of Transformer Models and Explicit Rule Constraints: Although this study implicitly smoothed the output trajectories to a certain extent by predicting action chunks, the Transformer decoder fundamentally remains highly reliant on a purely data-driven positional encoding mechanism to establish temporal relationships, lacking explicit constraints on continuous physical processes. When encountering extremely complex perturbations that fall out-of-distribution (OOD) from the training data, purely data-driven models carry the inherent risk of temporal mutations or local trajectory divergence. Future research will strive to introduce explicit temporal rule constraints into the model architecture to fundamentally bolster the system’s temporal stability in complex scenarios.

## 5. Conclusions

This study proposes a physical-parameter-decoupled imitation learning method for autonomous ultrasound scanning directed at diverse equipment instances. This method innovatively introduces task-space waypoint representation alongside a velocity-aware adaptive extraction mechanism, effectively overcoming the strong reliance on low-level kinematic parameters and the long-horizon error accumulation issues inherent in traditional imitation learning models. The empirical results demonstrate that this method achieved an 84% zero-shot deployment success rate on target-domain equipment that was entirely excluded from training and possessed objective physical parameter barriers. Furthermore, the average trajectory prediction error was constrained within 3.3 mm, and the system successfully maintained stable probe contact in a dynamic respiratory environment via admittance constant force control.

This study confirms that decoupling the clinician’s operative intent into a sparse sequence of waypoints endowed with global geometric constraints constitutes an effective pathway for realizing the reliable reuse of robotic skills across physical instances. This achievement not only significantly elevates the utilization efficiency of high-quality expert demonstration data but also provides crucial theoretical references and a technological paradigm for the future development of autonomous ultrasound diagnostic robots equipped with high system robustness and equipment compatibility. It holds promising potential to significantly alleviate medical resource shortages and propel the ubiquitous adoption of intelligent autonomous ultrasound diagnostics.

## Figures and Tables

**Table 1:** Quantification of Objective Physical Discrepancies Between Source and Target Equipment Instances.

Category of Physical Discrepancy	Parameter Definition & Notation	Physical Offset of Target Relative to Source	Impact of Physical Discrepancy
Global base translation	$\Delta P_{base}={\left[\Delta x,\Delta y,\Delta z\right]}^{T}$	Approx. [150, −80, 50] mm	Causes failure of spatial coordinate mapping
Global base rotation	$\Delta R_{base}={\left[\Delta \alpha ,\Delta \beta ,\Delta \gamma \right]}^{T}$	Approx. [0, 0, 4.5°]	Causes deviation in the normal contact angle of the end-effector probe
Link length tolerance	$\Delta a_{i},\Delta d_{i}$	1.2 mm (Average)	Triggers amplification of Inverse Kinematics (IK) calculation errors
Tool Coordinate System (TCP) offset	$\Delta T_{tool}$	Translation: ±20 mm/ Rotation: ±1.5°	Alters the actual ultrasound acoustic window plane and force control application point

**Table 2:** Comparison of Offline Trajectory Prediction Errors Across Different Network Architectures and Action Representations.

Network Backbone	Action Representation	Mean Error (mm)	SD (mm)	Max Error (mm)	95% CI of Mean (mm)	Significance (*p*-Value)
ACT	Incremental	11.2	4.2	14.3	[9.47, 12.93]	-
ACT	Waypoint (Ours)	3.3	1.4	4.6	[2.72, 3.88]	p<0.001
Diffusion Policy	Incremental	9.8	3.6	12.5	[8.61, 12.39]	-
Diffusion Policy	Waypoint (Ours)	3.1	1.2	4.2	[2.36, 3.84]	p<0.001

Note: The significance tests compare the differences in mean errors between the “Waypoint” and “Incremental” representations under the identical network backbone.

**Table 3:** Scanning success rate experiment.

Model	Source Domain Manipulator (95% CI)	Target Domain Manipulator (95% CI)
Incremental model	76% [56.6%, 88.5%]	64% [44.5%, 79.8%]
Waypoint-based model	92% [75.0%, 97.8%]	84% [65.3%, 93.6%]

**Table 4:** Experimental Results of *Z*-axis Force Control.

Evaluation Metrics	Source Domain Manipulator	Target Domain Manipulator
Average contact force	11.90 N	12.19 N
Standard deviation	0.72 N	0.69 N
Root mean square error	0.68	0.67
Percentage of Force Deviations within ±1.5 N	96.5%	97.3%

**Table 5:** Structural Ablation Results of Core System Modules.

Model Version	Action Representation	Velocity Planning & Physical Force Control	Task Success Rate (%)
Version 1 (Baseline)	Cartesian space increment	Enabled	76
Version 2	Fixed-precision waypoints (ϵ=0.0015)	Enabled	80
Version 3	Adaptive-precision waypoints	Disabled (Pure position servo only)	<10
Version 4 (Complete system)	Adaptive-precision waypoints	Enabled	92

**Table 6:** Ablation experimental results.

Precision Error			Error/mm	Time/Step	Success Rate/%
Fixed precision ϵ	0.007		9.8	300	56
	0.005		6.3	460	68
	0.003		5.1	580	72
	0.0015		4.5	670	80
Adaptive precision	ϵ_max_	ϵ_min_			
	0.008	0.006	7.4	340	64
	0.006	0.004	5.3	420	72
	0.004	0.002	4.2	460	88
	0.002	0.001	3.3	510	92

**Table 7:** Comparison of performance of different deep organ models.

Model	Position Error/mm	Success Rate/%	Region	Scanning Object
This study	3.3	92	Kidney	Human body
Reference [19]	7.90 ± 6.87	79.0	Spine	Human body
Reference [20]	2.6	72	Heart	Human body
Reference [21]	3.38 ± 0.45	96.7	Kidney	Phantom

## Data Availability

The data presented in this study are available on request from the corresponding author. For data requests, please contact: m_zy123456@163.com.

## Abbreviations

The following abbreviations are used in this manuscript:

ACT	Action Chunking Transformer
BMI	Body Mass Index
CVAE	Conditional Variational Auto-Encoder
D-H	Denavit-Hartenberg
HDMI	High-Definition Multimedia Interface
PI	Proportional-Integral
SD	Standard Deviation
TCP/IP	Transmission Control Protocol/Internet Protocol
USB	Universal Serial Bus
